# Direct Composite Resin Restoration for Midline Diastema Closure Using a Modified Matrix Technique: A Report of Two Cases

**DOI:** 10.7759/cureus.109916

**Published:** 2026-05-30

**Authors:** Gagan Kumar, Rekha Gupta, Shubhra Gill, Zeba Naaz

**Affiliations:** 1 Department of Prosthodontics, Maulana Azad Institute of Dental Sciences, New Delhi, IND

**Keywords:** aesthetic dentistry, composite resin, glue gun, hot-melt adhesive (hma), midline diastema

## Abstract

Midline diastema is commonly observed across various populations and may negatively affect dental aesthetics and patient confidence. Several treatment modalities are available for diastema closure, including direct and indirect composite veneers, porcelain laminate veneers, and full-coverage restorations. However, many patients seek conservative, economical, and minimally invasive aesthetic treatment options. This case series describes the clinical application of direct composite resin restorations for midline diastema closure using a modified transparent matrix technique. The technique utilizes a hot-melt thermoplastic adhesive (HMA) material as a transparent modified matrix to assist composite contouring and adaptation during restoration. The transparency of the matrix aids visualization during composite placement and finishing, while the customized form assists in achieving symmetry and proximal contouring. Direct composite resin restoration is a minimally invasive and cost-effective treatment option for the closure of midline diastema. In the presented cases, satisfactory aesthetic outcomes and patient acceptance were observed with preservation of natural tooth structure. Within the limitations of this case series, the modified HMA matrix-assisted composite technique appears to be a simple and practical approach for midline diastema closure. Further clinical studies comparing this technique with conventional matrix systems are recommended.

## Introduction

Facial aesthetics play an important role in patient perception and self-confidence. Midline diastema, defined as the spacing between the maxillary central incisors, is a common aesthetic concern encountered in dental practice [1]. Management depends on the underlying etiology, size of the diastema, occlusion, periodontal status, and patient expectations [2]. Although considered a natural variation in certain cultures, many individuals perceive it as an unattractive feature, leading to emotional and psychological distress [2]. This visible spacing may adversely affect a patient’s self-esteem and frequently motivates the pursuit of aesthetic correction [3].

Midline diastemas can arise from multiple etiological factors, including tooth-size discrepancies, abnormal frenum attachment, genetic influences, or oral habits, with spontaneous closure following frenectomy also being documented [4]. Increasing patient expectations for enhanced dental aesthetics, coupled with the contemporary focus on preserving natural tooth structure, has encouraged the development of restorative techniques capable of recreating lifelike anterior tooth morphology and closing diastemas conservatively [5]. Etiology-related factors, such as tooth-size disproportions and oral habits, must be carefully evaluated during treatment planning [6]. Based on the underlying cause and the extent of spacing, available management options include orthodontic treatment, which may provide conservative correction but often requires longer treatment duration and higher patient compliance. Indirect ceramic restorations offer excellent aesthetics and durability but involve greater cost and irreversible tooth preparation in some cases. Direct composite resin restorations are minimally invasive, economical, and can be completed in a single visit; however, achieving ideal proximal contour, symmetry, and emergence profile may be technique-sensitive [7]. Compared with other transparent elastomeric materials, these options differ markedly in aesthetics, durability, and cost-effectiveness [8]. Accurate identification and management of the etiological factors are essential for ensuring long-term stability following diastema closure [3].

Within the current trend of minimally invasive dentistry, direct composite resin restorations have gained substantial popularity due to their capacity to provide immediate, conservative, and natural-looking aesthetic results while preserving healthy tooth structure. Composite resins offer precise control over tooth shape, shade, and contour, enabling clinicians to achieve minimally invasive and practical outcomes [7].

This report describes the use of a modified hot-melt adhesive (HMA) matrix-assisted direct composite technique for midline diastema closure in two patients. The technique was used to assist composite contouring and matrix adaptation during restoration. Clinical procedures and immediate post-treatment outcomes are described.

## Case presentation

Case 1

A 25-year-old female patient presented to the outpatient department of the Department of Prosthodontics, Crown and Bridge, MAIDS, with a chief complaint of spacing between the maxillary central incisors. She reported social embarrassment and reduced self-confidence due to the aesthetic concern. Her medical history was non-contributory, with no relevant systemic illness.

**Figure 1:**
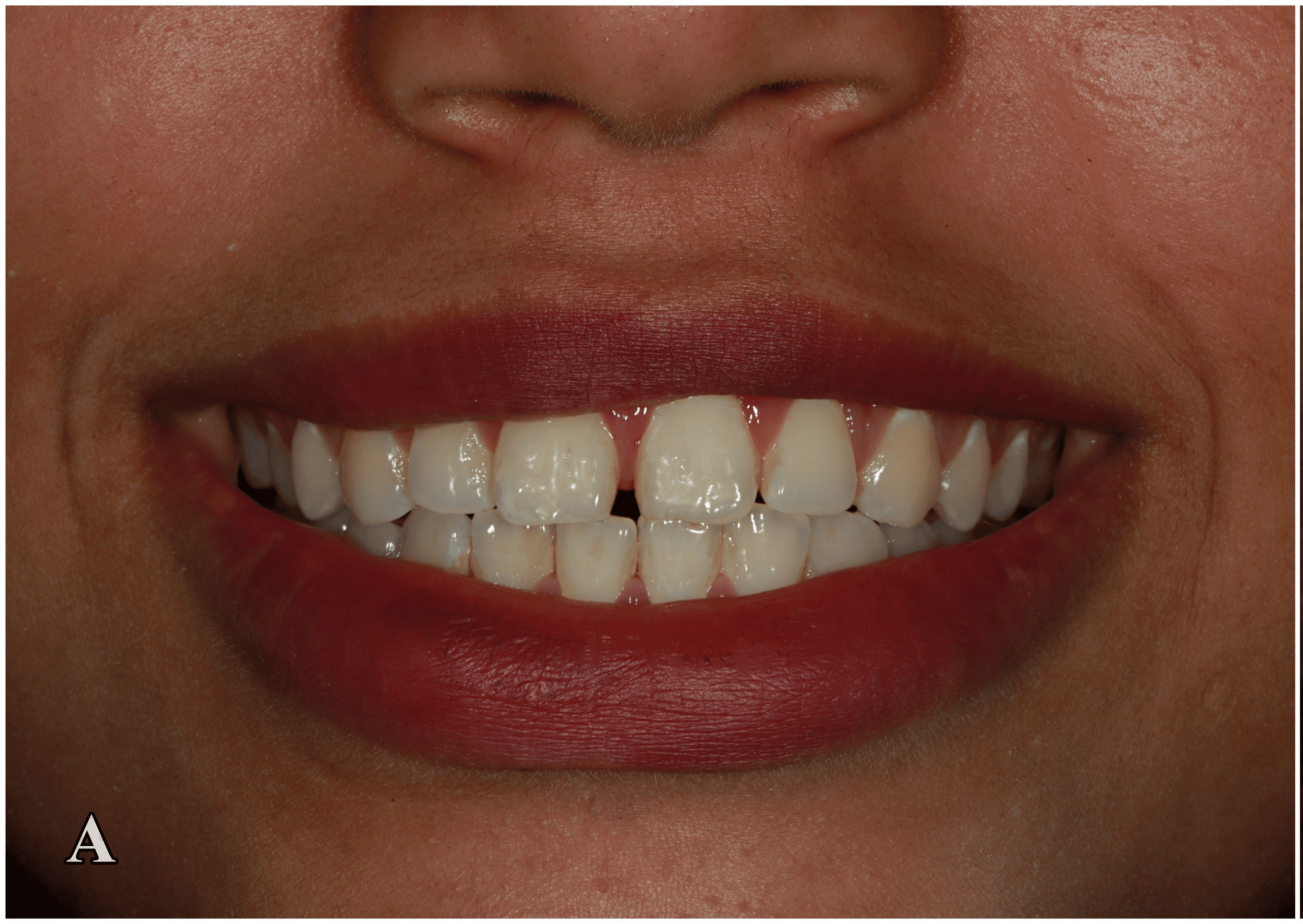
Preoperative intraoral photograph of Patient 1 showing midline diastema between the maxillary central incisors.

Intraoral examination revealed a midline diastema measuring approximately 2 mm between the maxillary central incisors (Figure 1). No traumatic occlusal contacts or functional interferences were observed. Periodontal examination revealed healthy gingival tissues with no evidence of periodontal pocketing or mobility. Vitality testing of the maxillary central incisors demonstrated a normal pulpal response. Frenum examination revealed no abnormal high frenum attachment. No parafunctional oral habits were reported. Periapical radiographic examination showed no periapical pathology or underlying osseous abnormalities. Based on the clinical findings and patient expectations, a modified matrix-assisted composite technique was planned for diastema closure. Treatment was completed over two clinical visits. The first visit included diagnostic impressions, cast preparation, wax-up procedures, and fabrication of the modified HMA matrix. The second visit involved clinical application of the matrix-assisted composite restoration.

**Figure 2:**
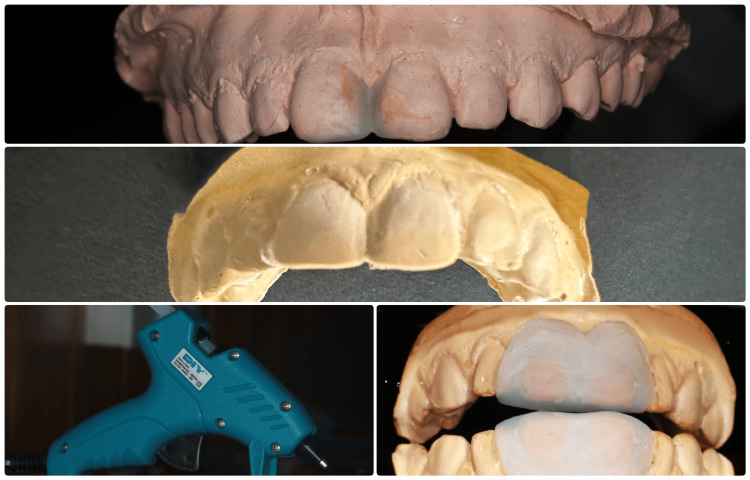
Diagnostic wax-up, duplicated cast preparation, and fabrication of the modified hot-melt adhesive matrix using an electric glue gun.

Primary impressions were made using elastomeric impression material, and diagnostic casts were prepared. A diagnostic wax-up was completed to replicate the planned final tooth morphology. The wax-up was duplicated using elastomeric putty and light-body materials to obtain a stone cast. Molten HMA was injected onto the duplicated cast using an electric glue gun to fabricate the modified matrix (Figure 2), which was subsequently trimmed and finished to obtain a thin, flexible adaptation that allowed accurate intraoral seating while maintaining adequate contour guidance during composite placement.

**Figure 3:**
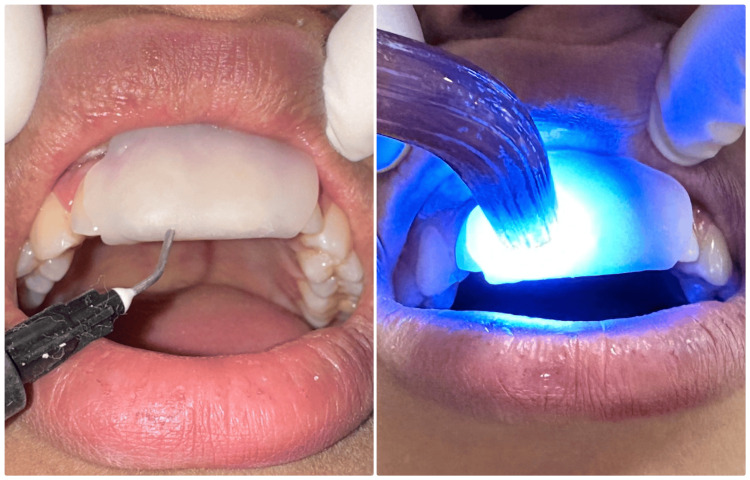
Intraoral placement of the hot-melt adhesive matrix and injection of flowable light-cured composite resin for diastema closure in Patient 1.

During the clinical appointment, relative isolation was achieved using cotton rolls and saliva ejectors, followed by shade selection. Enamel etching with 37% phosphoric acid was performed for 15 seconds, followed by rinsing for 20 seconds and gentle air drying. A bonding agent (Ivoclar Vivadent Te-Econom Bond) was applied and light-cured for 20 seconds. The HMA matrix was positioned over the tooth surface, and PTFE tape was placed interproximally to prevent composite overflow. Flowable composite resin (Ivoclar Tetric N-Flow) was injected through the buccal access opening to close the diastema (Figure 3).

**Figure 4:**
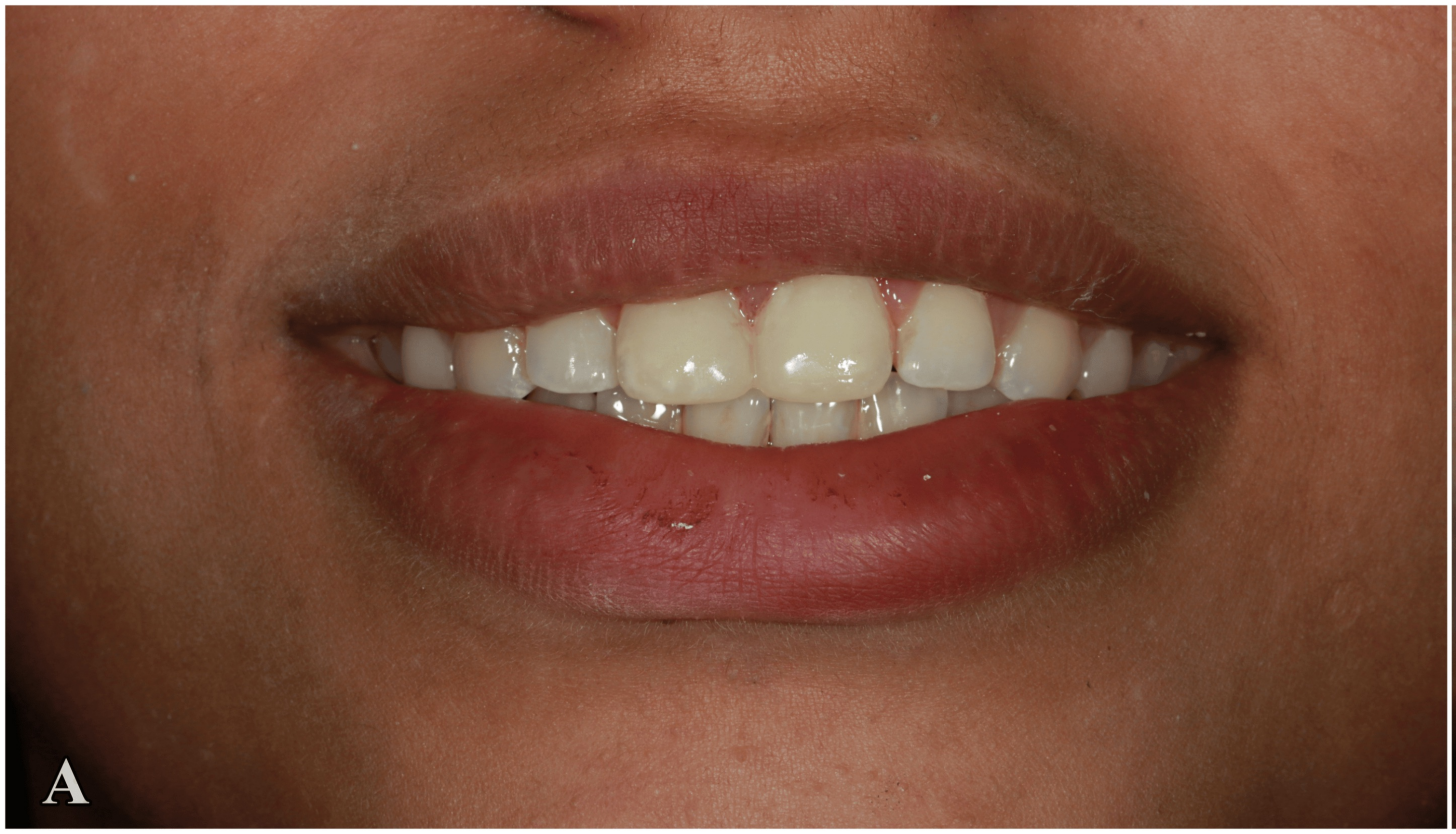
Postoperative intraoral photograph of Patient 1 after composite resin diastema closure.

**Figure 5:**
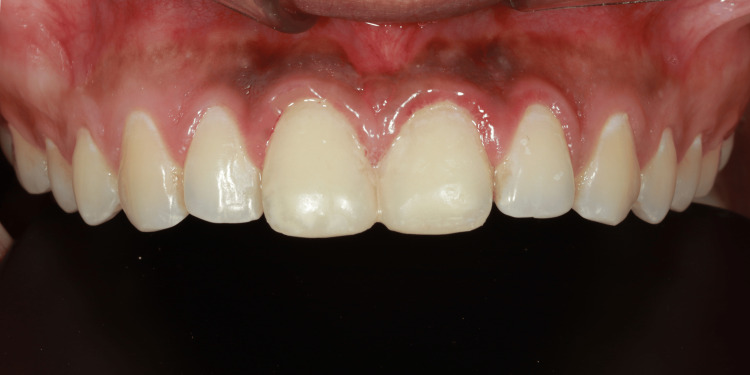
Postoperative close-up view demonstrating composite restoration and midline diastema closure in Patient 1.

After polymerization, finishing and polishing were performed using the Shofu Composite Polishing Kit. Postoperative photographs were recorded to document the clinical outcome (Figures 4, 5). No medications were prescribed. At the one-month follow-up, the restorations remained intact with satisfactory marginal adaptation and no postoperative sensitivity.

Case 2

A 27-year-old female patient presented with a chief complaint of spacing between the maxillary central incisors associated with aesthetic dissatisfaction during social interactions. Medical history was non-contributory.

**Figure 6:**
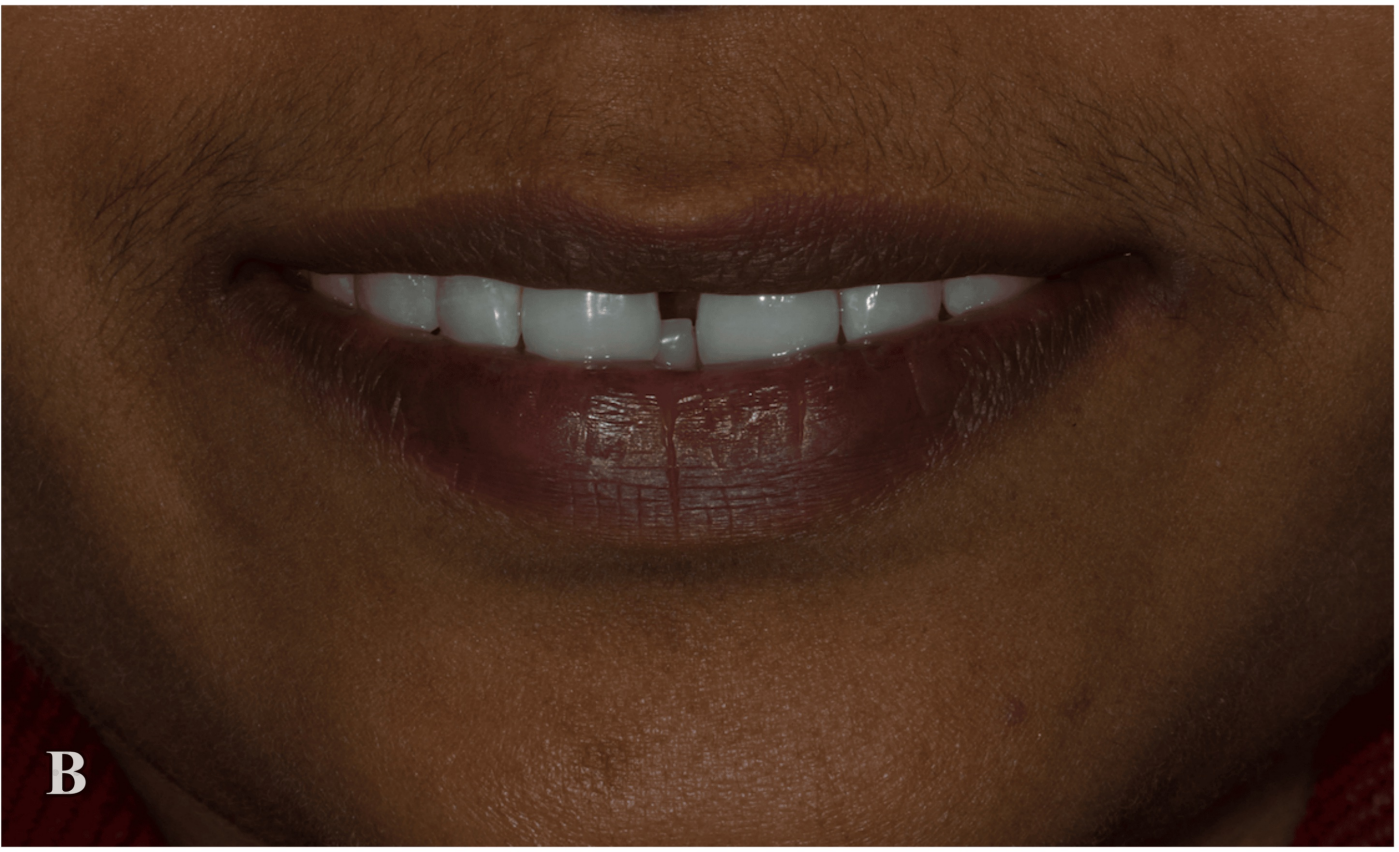
Preoperative photograph of Patient 2 showing diastema space.

Clinical examination revealed a midline diastema measuring approximately 2.5 mm between the maxillary central incisors (Figure 6). Periodontal and radiographic examinations showed no significant abnormalities. Vitality testing demonstrated normal pulpal response, and no traumatic occlusal contacts or parafunctional habits were identified.

**Figure 7:**
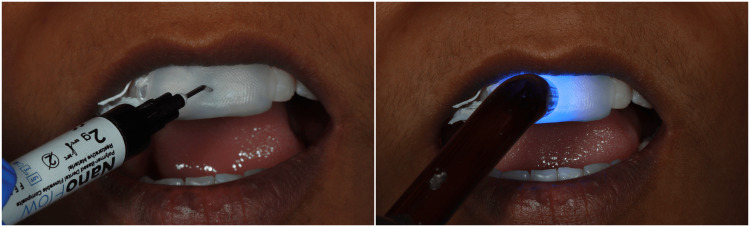
Placement of the hot-melt adhesive matrix and insertion of flowable light-cure composite resin onto teeth in Patient 2.

Following diagnostic impressions and wax-up procedures, a modified HMA matrix was fabricated in a manner similar to Case 1. Relative isolation was achieved using cotton rolls and saliva ejectors. Shade selection, etching, bonding, and placement of PTFE tape were subsequently performed. Flowable composite resin (Ivoclar Tetric N-Flow) was injected through the matrix to achieve diastema closure (Figure 7).

**Figure 8:**
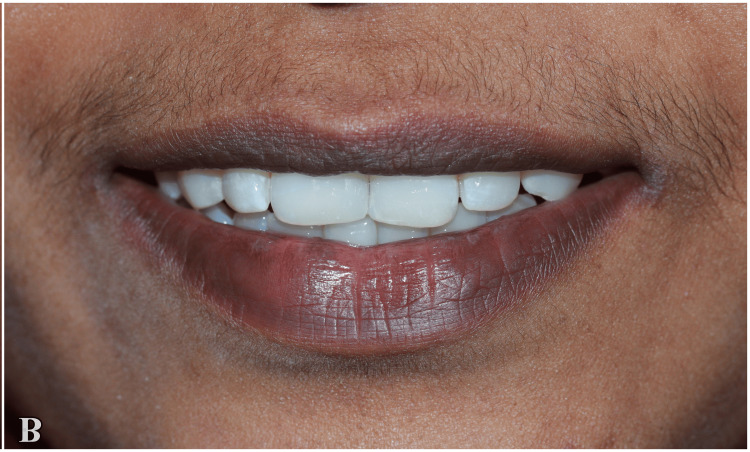
Post-restoration photograph of Patient 2.

**Figure 9:**
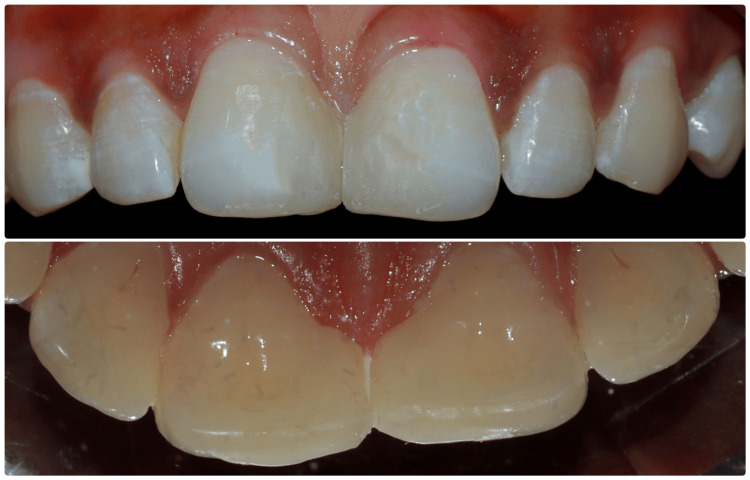
Post restoration close-up view of Patient 2.

Finishing and polishing procedures were completed using the Shofu Composite Polishing Kit. Postoperative photographs demonstrated satisfactory aesthetic correction (Figures 8, 9). Postoperative clinical evaluation demonstrated satisfactory symmetry, proximal contact adaptation, and emergence profile without visible black triangle formation. The patients expressed subjective satisfaction with the immediate postoperative aesthetic outcome; however, long-term follow-up was not available.

Consent and ethical considerations

Written informed consent was obtained from both patients for treatment and publication of clinical photographs. Ethical approval was waived as this report describes routine clinical procedures performed as part of standard patient care.

## Discussion

With the increasing emphasis on minimally invasive dentistry, direct composite resin restorations have become a widely used option for the aesthetic management of anterior spacing while preserving natural tooth structure. Compared with indirect ceramic restorations, direct composites are more economical, repairable, and can generally be completed in a single clinical visit [5].

Various matrix-assisted techniques have previously been described for composite diastema closure, including silicone index and putty matrix systems. Other techniques, such as Mylar strip matrices, freehand layering, and injection moulding approaches, have also been described for diastema closure, each with varying degrees of operator dependence and laboratory involvement. In the present report, a modified HMA matrix was used to assist composite contouring and restoration shaping during placement. The matrix provided sufficient light transmission for composite resin polymerization while assisting in proximal contour development and matrix adaptation.

The HMA matrix-assisted technique used in these cases allowed controlled composite placement and reduced the need for extensive finishing procedures. However, the technique requires additional laboratory procedures, including diagnostic wax-up and matrix fabrication, which may necessitate an additional clinical visit compared with direct freehand composite techniques. The HMA material used in the present technique was primarily based on ethylene-vinyl acetate, a thermoplastic copolymer commonly used in adhesive formulations. Similar thermoplastic adhesive systems have also been investigated in selected biomedical applications [9]. However, in the present technique, the HMA material was used only as a temporary external matrix aid during restoration and was removed immediately after composite resin polymerization. No adverse clinical reactions were observed in the present cases, although further investigations evaluating biocompatibility, dimensional stability, sterilization protocols, and long-term clinical safety are necessary before broader clinical application. The technique may serve as a practical and economical matrix-assisted approach for selected patients seeking conservative single-visit aesthetic rehabilitation.

Despite the advantages of direct composite restorations, certain limitations should be considered. Flowable composites may demonstrate polymerization shrinkage, potential staining over time, and lower fracture resistance compared with indirect ceramic restorations. In addition, long-term maintenance and periodic polishing may be required to preserve surface texture and aesthetics.

Limitations of this report include the small number of cases and a limited follow-up duration. Long-term clinical performance of the restorations could not be assessed comprehensively. Therefore, parameters such as color stability, marginal discoloration, fracture resistance, debonding, gingival response, wear, and maintenance requirements could not be adequately evaluated. Additional limitations include the absence of objective quantitative outcome assessment and a lack of comparative analysis with conventional matrix systems. Formal symmetry analysis, validated patient-reported outcome measures, and objective aesthetic assessment criteria were not performed. Further clinical studies with larger sample sizes and long-term follow-up are required to assess the reproducibility and long-term clinical performance of the technique.

## Conclusions

Within the limitations of this report, the modified HMA matrix-assisted composite technique was successfully used for short-term management of midline diastema in two clinical cases. The technique may assist composite contouring and matrix adaptation during restoration. However, further clinical studies with larger sample sizes, control comparisons, objective outcome assessment, and long-term follow-up are necessary before definitive conclusions regarding clinical effectiveness and long-term performance can be established.
